# Identification of Novel Mycobacterial Inhibitors Against Mycobacterial Protein Kinase G

**DOI:** 10.3389/fmicb.2018.01517

**Published:** 2018-07-12

**Authors:** Yuichi Kanehiro, Haruaki Tomioka, Jean Pieters, Yutaka Tatano, Hyoji Kim, Hisashi Iizasa, Hironori Yoshiyama

**Affiliations:** ^1^Department of Microbiology, Faculty of Medicine, Shimane University, Izumo, Japan; ^2^Department of Basic Medical Sciences for Nursing, Yasuda Women’s University, Hiroshima, Japan; ^3^Biozentrum, University of Basel, Basel, Switzerland; ^4^Department of Pharmaceutical Science, International University of Health and Welfare, Ohtawara, Japan

**Keywords:** eukaryotic-like serine/threonine kinase, PknG, macrophages, phagolysosome, anti-mycobacterial, chemotherapy

## Abstract

Protein kinase G (PknG) is a eukaryotic-like serine/threonine kinase that is expressed by *Mycobacterium tuberculosis* and promotes survival of mycobacteria in host macrophages by suppressing phagosome-lysosome fusion. Thus, compounds showing inhibitory activity against PknG are promising anti-mycobacterial agents. We therefore aimed to develop anti-mycobacterial agents by identifying new PknG inhibitors. A luciferase-based PknG kinase assay was used to screen potential inhibitors of PknG. We found that four compounds, namely AZD7762, R406, R406-free base, and CYC116, inhibited PknG activities. AZD7762, R406, and R406-free base promoted transfer of mycobacteria to lysosomes. These compounds also inhibited survival of *M. bovis* Bacillus Calmette–Guérin (BCG) inside human macrophages. Furthermore, R406 and R406-free base showed bactericidal activity against BCG in infected human macrophages without cytotoxicity. The PknG inhibitors identified in this study by the luciferase-based PknG kinase assay may be promising leads for the development of anti-mycobacterial agents.

## Introduction

Tuberculosis (TB) is one of the most important global health problems, with about 10 million new cases arising every year (Zumla et al., 2015). The virulence of *Mycobacterium tuberculosis* is linked to its capacity to survive within macrophages that are designed to destroy phagocytosed pathogens (Frieden et al., 2003). While in healthy persons, a functional immune system keeps intracellular bacteria in check, if the immune system of infected persons is impaired by, for example, infection with the human immunodeficiency virus or the administration of anti-inflammatory drugs or immunosuppressants, *M. tuberculosis* can rapidly spread and cause disease (Frieden et al., 2003). An additional problem is that strains resistant against current drugs are spreading globally (Matteelli et al., 2014), and therefore it is important to develop anti-TB agents that can efficiently target *M. tuberculosis*.

*Mycobacterium* spp., such as *M. tuberculosis* and *M. bovis* Bacillus Calmette–Guérin (BCG), survive and persistently infect in macrophages by escaping from the host lysosomal degradation pathway (Armstrong and Hart, 1975). A eukaryotic-like serine/threonine kinase, protein kinase G (PknG), was shown to play a pivotal role in blocking phagosome-lysosome fusion within infected macrophages (Walburger et al., 2004). Mycobacteria that are deficient in PknG are predominantly found within lysosomes compared with wild-type mycobacteria that reside in phagosomes (Walburger et al., 2004). In addition, when severe combined immunodeficiency mice are infected with *M. tuberculosis*, the mice infected with PknG-deficient bacteria are able to survive longer than those infected with wild-type bacteria (Cowley et al., 2004; Sundaramurthy et al., 2017), suggesting that suppression of phagosome-lysosome fusion by PknG allows mycobacteria to escape from lysosomal degradation and to grow within macrophages. Blocking PknG by use of the specific PknG kinase inhibitor AX20017, which targets the ATP binding site of the PknG kinase domain, resulted in the effective lysosomal delivery and death of internalized mycobacteria (Walburger et al., 2004; Scherr et al., 2007). PknG-inhibiting compounds discovered from flavonoids and aminopyrimidine derivatives were considered promising anti-mycobacterials (Anand et al., 2012).

It is important to increase the number of lead compounds that inhibit PknG activity. Additional PknG inhibitors were screened by measuring ATP consumption, based on kinase activity that transfers phosphate groups from ATP to substrates (Baki et al., 2007). The radiometric-based kinase assay for screening PknG inhibitors measured the incorporation of radioactive phosphate into myelin basic protein (MBP) (Koul et al., 2001). In this assay, MBP was incubated with γ^33^P-labeled ATP in the presence of PknG. We here present a luciferase-based PknG kinase assay that was used to search for PknG inhibitors that can effectively suppress intracellular mycobacterial growth.

## Materials and Methods

### Cell Lines and Bacterial Strains

THP-1 and J774.1 cell lines were cultured with RPMI 1640 medium (Sigma-Aldrich, St. Louis, MO, United States) supplemented with 10% fetal calf serum. The THP-1 cells were differentiated by stimulation with 50 nM phorbol 12-myristate 13-acetate (Sigma-Aldrich) for 2 days. *M. bovis* strain BCG-Tokyo 172 (BCG) was purchased from ATCC (Manassas, VA, United States). Green fluorescent protein (GFP)-expressing BCG (WT BCG) and PknG knockout BCG-GFP (ΔPknG BCG) were also used (Walburger et al., 2004). BCG was propagated in 7H9 mycobacterial medium (BD, Franklin Lakes, NJ, United States) supplemented with 10% Middlebrook ADC Growth Supplement (Sigma-Aldrich) and 0.05% Tween80 or 7H11 mycobacterial medium (BD) supplemented with 10% Middlebrook OADC Growth Supplement (Sigma-Aldrich) and 0.05% Tween80. The bacterial suspension was gently sonicated using a sonicator (UR-20P, Tomy, Tokyo, Japan) for 5 s and centrifuged at 150 × *g* for 5 min to eliminate bacterial clumps and was then used.

The protocols related to biosafety issues were approved by the institutional review board of the Shimane University. Each study was conducted under the rules for preventing dispersal of living modified organism. The living modified organisms were handled inside a biosafety cabinet at a level 2 or level 3 facility.

### GST-PknG Fusion Protein Purification

The *pknG* gene was amplified by PCR using KOD-Plus polymerase (Toyobo, Osaka, Japan) from *M. tuberculosis* H37Ra (ATCC 25177) genomic DNA using specific primers (FW: 5′-GGCCAAGCGTCAGAGACCGAACGTTCGGG-3′, RV: 5′-TTAGAACGTGCTGGTGGGCCGGACCTTG-3′). For the thermal cycling, initial denaturation at 94°C for 3 min was followed by 35 cycles of denaturation at 94°C for 30 s, annealing at 55°C for 30 s, and extension at 68°C for 3 min. Amplified *pknG* gene was inserted into pGEX-3X vector (GE Healthcare, Little Chalfont, United Kingdom) to create a *GST*-*pknG* fusion gene (Smith and Johnson, 1988), which was expressed in *E. coli* strain BL21 (GE Healthcare) and induced by 0.1 mM IPTG treatment at 25°C.

Bacterial cell pellets were suspended with cold phosphate buffered saline (PBS), disrupted by sonication (ON: 30 s/OFF: 30 s × 10) (Bioruptor, UCD-200, Cosmo Bio, Tokyo, Japan) and then dissolved with 1% Triton-X100. The mixture was clarified by a 5 min centrifugation at 13,400 × *g*. The supernatant was mixed with glutathione-sepharose 4B (GE Healthcare) beads and incubated for 30 min at 4°C. The beads were washed five times with cold PBS by centrifugation (2,300 × *g*, 5 min). The washed beads were then suspended with lysis buffer (20 mM reduced glutathione, 50 mM Tris–HCl, pH 8.0) and incubated for 5 min on ice. Supernatants obtained by centrifugation at 2,300 × *g* for 5 min were subjected to SDS–PAGE to verify the purified GST-PknG.

### Luciferase-Based PknG Kinase Assay

A luciferase-based PknG kinase assay was established with minor modification from the previously reported method (Baki et al., 2007). The test compounds dissolved with dimethyl sulfoxide (DMSO) were diluted with double-distilled H_2_O. The reagents composed of 20 μL of mixture (10 μM test compound, 1 μM GST-PknG, and 20 μL of kinase reaction buffer [5 μM MBP (Funakoshi, Tokyo, Japan), 50 mM Tris–HCl (pH 7.6), 1 μM ATP, 1 mM dithiothreitol, 10 mM MnCl_2_, 250 μg/mL bovine serum albumin]) were added in a white 96-well plate (Thermo Fisher Scientific, Waltham, MA, United States). The mixtures were incubated in the modified kinase reaction buffer (Koul et al., 2001) at 30°C for 30 min and then mixed with 40 μL of Kinase-Glo reagent (Promega, Madison, WI, United States). After incubating at room temperature for 10 min, the luminescence signal was measured by a DTX-880 multi-mode plate reader (Beckman Coulter, Brea, CA, United States).

We used 1 μM of ATP to screen the PknG inhibitors. The level of ATP used was 1,000-fold lower than the level of the intracellular ATP (Beis and Newsholme, 1975). However, the linear relationship between the concentration of ATP and the intensity of the luminescent signal could be observed in the ATP concentrations between 0.1 and 16 μM, which was similar to the original assay (Baki et al., 2007).

### Kinase Inhibitors

AZD7762 (3-(carbamoylamino)-5-(3-fluorophenyl)-*N*-[(3*S*)-piperidin-3-yl] thiophene-2-carboxamide) is a checkpoint kinase (Chk) 1/2 inhibitor and exhibits anti-tumor activity by inducing cell cycle arrest (Zabludoff et al., 2008). R406 ((6-[[5-fluoro-2-[(3,4,5-trimethoxyphenyl) amino]-4-pyrimidinyl] amino]-2,2-dimethyl-2H-pyrido[3,2-b]-1,4-oxazin-3(4H)-one)) and R406-free base (R406f) are human spleen tyrosine kinase (Syk) inhibitors and used as lead compounds of drugs for rheumatoid arthritis (Braselmann et al., 2006). CYC116 (4-methyl-5-[2-(4-morpholinophenylamino) pyrimidin-4-yl] thiazol-2-amine) is an Aurora kinase A/B inhibitor and shows anti-tumor activity by repression of cell division (Wang et al., 2010). These inhibitors were purchased from Sigma-Aldrich. These inhibitors were dissolved with DMSO and further diluted with each buffer or medium.

### Quantitative Inhibitory Assay for Inhibitors Against PknG Kinase

The luciferase-based PknG kinase inhibitory assay was performed using a kinase inhibitor library (Kinase inhibitor library I, Sigma-Aldrich) and PknG inhibitor (AX20017, Merck Millipore, Billerica, MA, United States). The inhibition rate of kinase activity by various compounds (%) was calculated as follows: Inhibition (%) = 100 × [relative light intensity units (RLU) raw data of sample – RLU positive control] / (RLU negative control – RLU positive control). All data were plotted in terms of an inhibition rate of AX20017 equal to 100%.

The half-maximal inhibitory concentration (IC50) was calculated from the sigmoidal curve for the GST-PknG kinase inhibition rate. The RLU values of GST-PknG and DMSO alone were used as a positive and a negative control, respectively.

### Direct Bactericidal Assay by Mycobacterial Growth

Bacillus Calmette–Guérin at 1 × 10^5^ colony forming units (CFUs)/well was incubated with 10 μM of kinase inhibitors at 37°C for 24 h. The bacterial suspension was diluted serially in 7H9 medium with 10% Middlebrook ADC Growth Supplement and 0.05% Tween 80. A total of 10 μl of each diluent were plated onto 7H11 agar plates containing 10% Middlebrook OADC Growth Supplement and 0.05% Tween 80. The plates were cultured for 3–4 weeks at 37°C and CFUs were counted.

The ability of the compounds to inhibit bacterial growth was assayed using alamarBlue cell viability reagent (Thermo Fisher Scientific) to measure the reducing viability of bacteria. Mycobacteria adjusted to an OD_600_ of 0.05 were incubated with 7H9 medium containing 10% OADC and fourfold serial dilutions (from 128 to 0.03 μM) of kinase inhibitors for 6 days at 37°C. Rifampicin was used as a control to show inhibition. After incubation, 20 μL of alamarBlue cell viability reagent was added to the bacterial suspension and incubated for 4 h at 37°C. The fluorescent signal (excitation: 535 nm/emission: 595 nm) was measured by a DTX-880 plate reader.

### Immunostaining

J774.1 cells on culture slides were infected with bacteria at a multiplicity of infection (MOI) of 10 for 30 min and then incubated with 10 μM of inhibitors for 90 min. Cells fixed with methanol for 4 min at -20°C were stained with Alexa Fluor 594 conjugated antibody against CD107a, known as lysosomal-associated membrane protein-1 (LAMP1) (Cosmo Bio), for 45 min at room temperature (Chen et al., 1985). Slides were observed using an FV1000-D laser scanning confocal microscope (Olympus, Tokyo, Japan). One hundred randomized events were analyzed for each drug, which were repeated three times and statistically analyzed. The rate of lysosomal transfer (%) was calculated as follows: lysosomal transfer (%) = 100 × (number of bacteria overlapping LAMP-1 / total number of analyzed bacteria.)

### Phagocytosis Assay

Phagocytosis assay was performed as previously reported with some modifications (Daigneault et al., 2010). J774.1 or THP-1 macrophages were seeded at 1.0 × 10^5^ cells into each well of a 96-well plate and incubated with fourfold serial dilutions of kinase inhibitors (from 128 to 0.03 μM) for 2 h at 37°C. Then, red fluorescent latex beads with a mean diameter of 2 μm (L3030; Sigma-Aldrich) were opsonized by 10% human serum (Cosmo Bio) for 30 min at 37°C. The opsonized beads were added to cells at an MOI of 10. The cells were then incubated for 2 h with different concentrations of inhibitors. Cells were washed twice with PBS, and red fluorescence (excitation, 535 nm/emission, 595 nm) was analyzed by a FACS Calibur flow cytometer (BD). We used 10 μM of cytochalasin D (Sigma-Aldrich) as a control, which completely inhibits actin polymerization (Cooper, 1987).

The fluorescence was compared with controls in 5,000 events. The phagocytosis rate (%) was calculated according to the following formula: phagocytosis (%) = 100 × [fluorescent cells (sample—negative control) / fluorescent cells (positive control – negative control)]. Positive and negative controls were the addition of fluorescent beads and DMSO alone, respectively.

### MTT Assay

To measure the cytotoxic effects of the kinase inhibitors, 3-(4,5-dimethylthiazol-2-yl)-2,5-diphenyltetrazolium bromide (MTT) was used, because mitochondrial succinate dehydrogenase in living cells reduces MTT to form insoluble formazan products (Mosmann, 1983). Cells were seeded at 1.0 × 10^5^ cells to each well of a 96-well plate and incubated with serial concentrations of kinase inhibitors for 24 h at 37°C. A total of 20 μl of MTT reagent adjusted to 5 μg/mL was added to each well. The plate was shaken for 10 min and incubated in a dark condition for 1 h at 37°C. Excess dye was removed and 200 μL of DMSO was added to the plate. An absorbance of 550 nm was measured by a DTX-880 plate reader.

Staurosporine was used as a control drug showing cytotoxicity, because the drug is a potent, cell permeable protein kinase C inhibitor and induces cell apoptosis (Couldwell et al., 1994).

The cell survival rate (%) was calculated by the formula: cell survival (%) = 100 × [(absorbance of kinase inhibitors treated cells – absorbance of solvent) / (absorbance of DMSO treated cells – absorbance of solvent)].

### Mycobacterial Survival Assay

Differentiated THP-1 cells were infected with BCG at an MOI of 10 for 2 h and then incubated with 10 μM of each inhibitor for an additional 2 h (Chaurasiya and Srivastava, 2009). Infected macrophages were washed once with PBS and incubated with amikacin (20 μM) and each inhibitor (10 μM) for 24 h at 37°C. Macrophages were washed three times with warm media and lysed with 200 μL of 0.05% SDS solution. The reducing activity of bacteria was measured by alamarBlue cell viability assay. The lysate was mixed with 20 μL of alamarBlue cell viability reagent and incubated for 12 h at 37°C. The fluorescent signal was measured by a DTX-880 plate reader (excitation: 535 nm/emission: 595 nm). Colony forming assay was performed at the same time as described in the direct bactericidal assay.

### Statistical Analysis

Data are shown as the mean ± SEM. The statistical significance between means of individual datum points was determined by one-way analysis of variance (ANOVA) test. Data are means ± standard deviations from three independent experiments. A *P*value of less than 0.05 was regarded as significant. Correlation between data is indicated in the plots as the *R*^2^ Pearson correlation coefficient.

## Results

### Screening of PknG Inhibitors by Luciferase-Based PknG Kinase Assay

**FIGURE 1:**
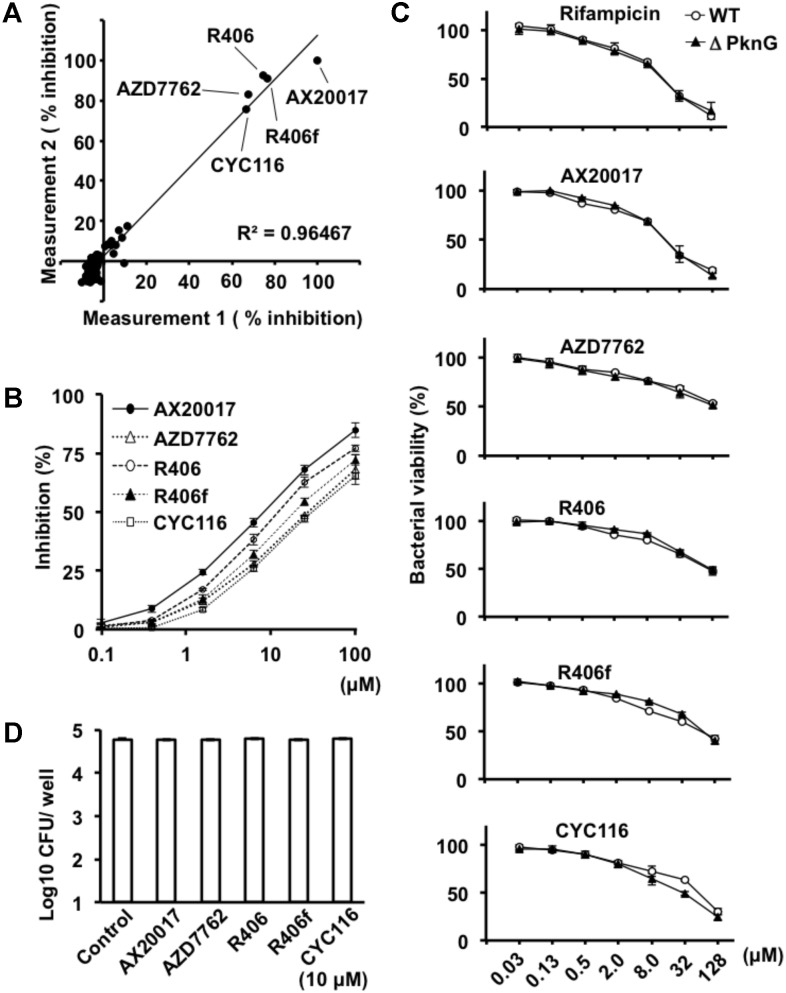
Effect of PknG inhibitors on kinase activity and bacterial survival. **(A)** Inhibition of PknG by a luciferase-based PknG activity assay. Kinase inhibition assays were performed using compounds from a kinase inhibitor library. The kinase reaction was performed using 5 μM of MBP as a substrate, 1 μM of ATP, 1 μM of GST-PknG, and 10 μM of compound per reaction. The experiment was repeated twice. The initial results were plotted on the horizontal axis and the second results were on the vertical axis. Correlation between the data is indicated in the plot as the (*R*^2^) Pearson correlation coefficient. Filled black circle indicates % Inhibition of PknG activity. **(B)** Inhibition of GST-PknG activity by different compounds. Kinase inhibitory assay was performed using fourfold serial dilutions of compounds in the range from 0.1 to 100 μM. **(C)** Dose-dependent inhibition of bacterial growth and metabolism. Mycobacteria were cultured with fourfold serial dilutions of compounds in the range from 0.03 to 128 μM for 6 days at 37°C. After the culture, 20 μL of alamarBlue reagent was added to the culture and incubated for 4 h at 37°C. Percent viability of WT (white circle) and ΔPknG (filled triangle) was measured by fluorescence intensity. **(D)** Effect of kinase inhibitors on mycobacterial survival. *Mycobacterium bovis* BCG/Tokyo strain was incubated at 1 × 10^5^ CFUs/well with 10 μM of each kinase inhibitor at 37°C for 24 h. Bacteria were cultured and CFUs were determined after 3–4 weeks. White rectangle indicates CFU/well. WT: WT BCG, ΔPknG: ΔPknG BCG, R406f: R406-free base.

To identify PknG inhibitors, we employed a luciferase-based activity assay that is based on residual ATP levels after PknG-induced MBP phosphorylation (Koul et al., 2001). We screened a library of 80 kinase inhibitory compounds (**Supplementary Table S1**). The percent inhibition in the approximation curve was 1.058, and the *R*^2^ correlation coefficient of the plot was 0.9647. The experiments were performed twice for each compound according to the previous protocol reported by Baki et al. (2007). The two independent experiments showed reproducible data. Among the active compounds, AZD7762, R406, R406f, and CYC116 showed a level of inhibition exceeding 50% relative to the inhibition of PknG by AX20017 (**Figure 1A** and **Supplementary Table S1**), which were used for further investigation as candidates for anti-mycobacterial agents. The inhibitory effect was 75.2% by AZD7762, 83.8% by R406, 83.6% by R406f, and 71.2% by CYC116. The IC50 values of AX20017, AZD7762, R406, R406f, and CYC116 were 5.49, 30.3, 7.98, 16.1, and 35.1 μM, respectively (**Figure 1B**). These inhibitors did not show any inhibitory effect on the luciferase activity at the concentrations used in our assays (**Supplementary Figure S1**).

### Effect of Kinase Inhibitors on Bacterial Growth

To analyze the effect of each kinase inhibitor on bacterial growth, WT BCG, and ΔPknG BCG were incubated with serial concentrations of the kinase inhibitors. Bacterial growth was evaluated using alamarBlue reagent. The effect of each kinase inhibitor on bacterial growth was similar (**Figures 1C,D**). The number of CFUs of the BCG treated with 10 μM of each PknG inhibitor for 24 h at 37°C was almost identical (**Figure 1D**).

### Phagosome-Lysosome Fusion Analysis in BCG-Infected Macrophages

**FIGURE 2:**
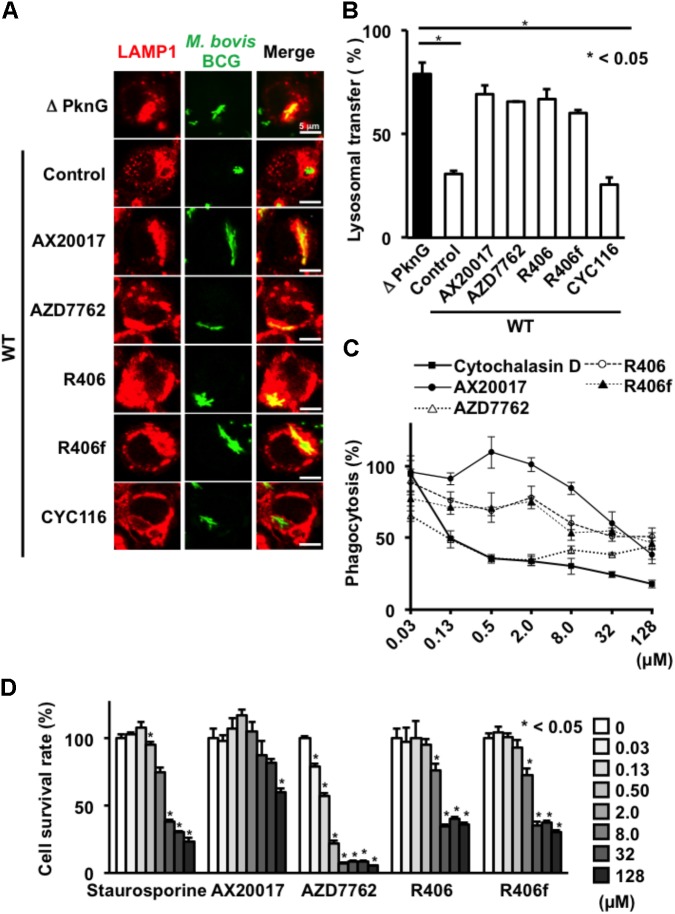
Effect of PknG inhibitors on phagocytosis and viability in murine macrophages. **(A)** Phagosome-lysosome fusion in BCG-infected macrophages. J774.1 cells were infected with bacteria at an MOI of 10 for 30 min, then incubated with 10 μM of PknG inhibitors for 90 min. Cells were fixed, stained with a lysosomal marker, and observed by a confocal laser scanning microscope. **(B)** Percentage of lysosomal transfer of WT BCG in the presence of 10 μM of each inhibitor. One hundred bacteria were counted in each experiment. The number of transferred bacteria observed for ΔPknG BCG (black bar) was the bacterial number subjected to maximal inhibition (79%). **(C)** Phagocytotic activity of murine macrophages treated with each compound. J774.1 cells were incubated with fourfold serial dilutions of kinase inhibitors from 0.03 to 128 mM for 2 h. Opsonized beads containing the same concentration of the test compound were added at an MOI of 10 and cultured. After 2 h, the fluorescence intensity was measured. **(D)** Cytotoxicity of each compound. Serially diluted compound was added to J774.1 cells and MTT assay was performed after 24 h. The statistical significance between means of individual datum points was determined by one-way ANOVA test. ^∗^*P* < 0.05. WT: WT BCG, ΔPknG: ΔPknG BCG, control: medium alone, R406f: R406-free base.

To evaluate the consequences of PknG inhibition on phagosome-lysosome fusion, J774.1 macrophages were infected with GFP-expressing BCG. After infection, cells were stained with lysosomal LAMP1 antibody. Intracellular BCG was counted (*n* = 100) by confocal microscopy. LAMP1 signals overlapping with GFP signals were regarded as phagosome-lysosome fusion positive. Fusion between intracellular WT BCG-containing phagosomes and lysosomes was 31% in the absence of inhibitors. However, the frequency of fusion increased to about 79% when cells were infected with ΔPknG BCG (**Figures 2A,B**). Importantly, after infection of WT BCG, the compounds AZD7762, R406, and R406f increased the frequency of fusion between the mycobacteria and lysosomes, whereas CYC116 failed to induce lysosomal delivery of internalized mycobacteria (**Figures 2A,B**).

### Effect of Novel PknG Inhibitors on Macrophages

**FIGURE 3:**
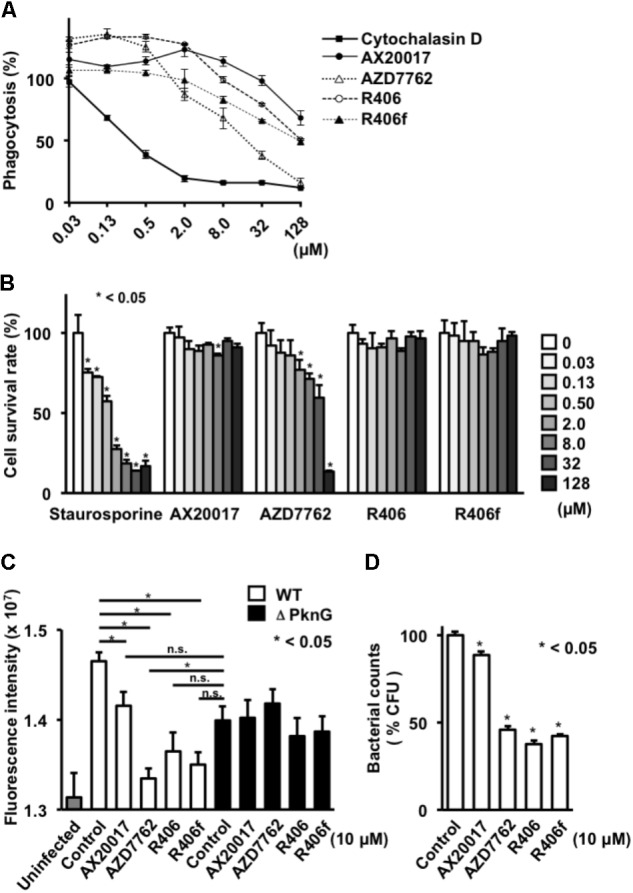
Effect of PknG inhibitors on phagocytosis, cell viability, and intracellular survival of bacteria in human macrophage. **(A)** Phagocytotic activity of differentiated THP-1 cells. Cells were incubated with fourfold serial dilutions of each compound from 0.03 to 128 mM for 2 h. Opsonized beads containing the same concentration of the test compound were added at an MOI of 10 and cultured. The fluorescence intensity was measured after 2 h. **(B)** Cytotoxicity of each compound. Serially diluted compound was added to the differentiated THP-1 cells. MTT assay was performed after 24 h. **(C)** Survival of mycobacteria in human macrophage after treatment with PknG inhibitors. THP-1 macrophages infected with WT BCG or ΔPknG BCG were treated with 10 μM of each compound. After 2 h incubation, infected macrophages were washed and incubated with amikacin (20 μM) in the presence of inhibitors. And each compound (10 μM) was added for another 24 h at 37°C. The macrophages were washed, lysed, and added with 20 μL of alamarBlue reagent. The mixtures were incubated for 12 h at 37°C. Fluorescence intensity of WT BCG (white bar), ΔPknG BCG (black bar), and null (gray bar) infected macrophages were measured. **(D)** Effect of PknG inhibitors on survival of mycobacteria in macrophages. Human THP-1 macrophages infected with BCG were treated with 10 μM of each compound. Infected THP-1 cells were lysed and serially diluted. A total of 10 μl of each diluent were spotted onto an agar plate. After incubation of the plates, the emerged bacterial colonies were counted. ^∗^*P* < 0.05. WT: WT BCG, ΔPknG: ΔPknG BCG, control: medium alone, R406f: R406-free base.

To assess the effect of the identified PknG inhibitors on macrophages, we examined the phagocytic activity, survival ratio, and bactericidal activity of macrophages incubated with different inhibitors (**Figures 2**, **3**).

To analyze phagocytosis, differentiated THP-1 cells were treated with each PknG inhibitor, and then fluorescent beads were added to the culture to evaluate the phagocytic ability. Phagocytic activity was slightly decreased at high concentrations of AX20017 and R406 and significantly impaired at 8 μM of R406f and AZD7762 (**Figure 3A**). We performed the same experiment using J774.1 cells. A significant reduction of phagocytic activity was observed for each kinase inhibitor except AX20017 (**Figure 2C**).

To examine cytotoxicity of the PknG inhibitors, the survival ratio of macrophages was measured by MTT assay. No cytotoxicity was observed on differentiated THP-1 macrophages treated with R406 or R406f, similar to treatment with AX20017. However, low levels of cytotoxicity were detected after 24 h in the presence of AZD7762 (**Figure 3B**). On the other hand, cytotoxicity of PknG inhibitors was more strongly observed on murine J774.1 cells (**Figure 2D**). Not only AZD7762, but R406 and R406f also showed cytotoxicity at concentrations higher than 2 μM (**Figure 2D**).

To evaluate bactericidal activity in human macrophages, differentiated THP-1 cells infected with WT BCG or ΔPknG BCG were incubated with PknG inhibitors. All PknG inhibitors reduced the growth or metabolic activity of WT BCG in human macrophages, that was not observed for mycobacteria lacking PknG, although to a different degree as observed in murine J774.1 macrophages (Walburger et al., 2004). Inhibition of bacterial growth was assessed by bacterial metabolic activity analysis as well as colony enumeration (**Figures 3C,D**). These results showed that the PknG inhibitors identified by the luciferase-based kinase assay reduced the growth or survival of BCG in a human macrophage cell line.

## Discussion

Isoniazid, rifampicin, pyrazinamide, and ethambutol have been used as anti-mycobacterial agents. However, it is very difficult to eradicate mycobacteria from every infected person, because existing antibiotics do not have strong potency against slowly proliferating mycobacteria. Moreover, intracellular mycobacteria are sequestrated from immune surveillance. Multidrug-resistant mycobacteria are also emerging. Therefore, the development of novel anti-mycobacterial drugs is strongly required.

In this work, compounds exhibiting PknG inhibition were screened using a luciferase-based PknG kinase assay. The discovered compounds R406 and R406f promoted fusion between mycobacteria-containing phagosomes and lysosomes. Both compounds showed cytotoxicity toward J774.1 cells at the concentration used for the phagosome-lysosome fusion assay, and reduced phagocytosis by THP-1 cells at the concentration used to measure intracellular BCG survival (**Figures 2**, **3**). Therefore, it is unclear whether these compounds change the fate of intracellular BCG via their effect on PknG activity or by their effects on other targets in the macrophages and the mycobacteria. However, CYC116, the other PknG inhibitor, did not enhance phagosome-lysosome fusion (**Figure 2B**). Another kinase inhibitor, AZD7762, which also inhibits Chk1/2, showed slight toxicity to macrophages (**Figure 3B**).

*Mycobacterium tuberculosis* has 11 serine-threonine protein kinases: PknA, PknB, PknC, PknD, PknE, PknF, PknG, PknH, PknJ, PknK, and PknL. These kinases are involved in a number of processes within *M. tuberculosis*, including growth, metabolism, and evasion from lysosomal degradation (Chao et al., 2010). Inhibitors against PknA and PknB retarded mycobacterial growth, and inhibitors against PknG suppressed phagosome-lysosome fusion (Székely et al., 2008). Moreover, sclerotiorin derived from *Penicillium frequentans* showed an inhibitory effect on PknG from *M. tuberculosis* (Chen et al., 2017). Sclerotiorin impaired mycobacterial survival in infected macrophages, but did not show direct inhibition of cultured mycobacterial growth. However, as happens many times, some compounds cannot be used on patients because of their side effects. Thus, it still worthwhile to develop novel compounds that inhibit serine-threonine protein kinases (Anand et al., 2012; Sipos et al., 2015).

The here used luciferase-based PknG kinase assay is a powerful tool for screening novel PknG inhibitors. We showed that R406 enhanced phagosome-lysosome fusion in addition to PknG inhibition (**Figure 2**). R406, R406f, and R788 (fostamatinib), a precursor of R406, are Syk kinase inhibitors (Braselmann et al., 2006; Weinblatt et al., 2010). Because R788 is already orally administered to patients suffering from rheumatoid arthritis, it should be possible to develop orally administrable anti-mycobacterial drugs by selecting drugs more specific to PknG than to Syk.

We used a modified luciferase based-kinase assay to identify inhibitors of PknG activity (Baki et al., 2007). Several PknG inhibitors were recently identified by pharmacophore-based virtual screening using the crystal structure of PknG (Singh et al., 2015). Because the amino acid sequence of PknG is identical between *M. bovis* BCG and *M. tuberculosis*, identification of PknG inhibitors having strong anti-BCG activity may also be relevant for finding drugs to combat *M. tuberculosis*. Our study showed one way to develop drugs targeting mycobacteria that are difficult to eradicate. However, there are many differences between mycobacteria in terms of bacterial toxicity and immunological response against the host. BCG lacks several genes required for the full pathogenicity of *M. tuberculosis* (Behr et al., 1999). The current observations should be pursued with the use of *M. tuberculosis* in the near future.

## Author Contributions

YK, YT, and HY conceived and designed the experiments. YK and YT performed the experiments. YK, HK, and HI analyzed the data. HI and YX wrote the manuscript. HT and JP reviewed the manuscript and supervised the research. All authors read and approved the final manuscript.

## Conflict of Interest Statement

The authors declare that the research was conducted in the absence of any commercial or financial relationships that could be construed as a potential conflict of interest.

## Supplementary Material

FIGURE S1 Effect of different compounds on luciferase activity. Luciferase activity was examined using 1 μM of ATP, fourfold serial dilutions of compounds in the range from 0.03 to 128 μM. R406f: R406-free base.

TABLE S1 Inhibitory activity of each PknG inhibitor by luciferase-based PknG kinase assay.

## References

[B1] Anand N. Singh P. Sharma A. Tiwari S. Singh V. Singh D. K. (2012). Synthesis and evaluation of small libraries of triazolylmethoxy chalcones, flavanones and 2-aminopyrimidines as inhibitors of mycobacterial FAS-II and PknG. *Bioorg. Med. Chem.* 20 5150–5163. 10.1016/j.bmc.2012.07.009 22854194

[B2] Armstrong J. A. Hart P. D. (1975). Phagosome-lysosome interactions in cultured macrophages infected with virulent tubercle bacilli. Reversal of the usual nonfusion pattern and observations on bacterial survival. *J. Exp. Med.* 142 1–16. 10.1084/jem.142.1.1 807671 PMC2189870

[B3] Baki A. Bielik A. Molnár L. Szendrei G. Keserü G. M. (2007). A high throughput luminescent assay for glycogen synthase kinase-3 beta inhibitors. *Assay Drug Dev. Technol.* 5 75–83. 10.1089/adt.2006.029 17355201

[B4] Behr M. A. Wilson M. A. Gill W. P. Salamon H. Schoolnik G. K. Rane S. (1999). Comparative genomics of BCG vaccines by whole-genome DNA microarray. *Science* 284 1520–1523. 10.1126/science.284.5419.1520 10348738

[B5] Beis I. Newsholme E. A. (1975). The contents of adenine nucleotides, phosphagens and some glycolytic intermediates in resting muscles from vertebrates and invertebrates. *Biochem. J.* 152 23–32. 10.1042/bj1520023 1212224 PMC1172435

[B6] Braselmann S. Taylor V. Zhao H. Wang S. Sylvain C. Baluom M. (2006). R406, an orally available spleen tyrosine kinase inhibitor blocks fc receptor signaling and reduces immune complex-mediated inflammation. *J. Pharmacol. Exp. Ther.* 319 998–1008. 10.1124/jpet.106.109058 16946104

[B7] Chao J. Wong D. Zheng X. Poirier V. Bach H. Hmama Z. (2010). Protein kinase and phosphatase signaling in *Mycobacterium tuberculosis* physiology and pathogenesis. *Biochim. Biophys. Acta* 1804 620–627. 10.1016/j.bbapap.2009.09.008 19766738

[B8] Chaurasiya S. K. Srivastava K. K. (2009). Downregulation of protein kinase C-alpha enhances intracellular survival of *Mycobacteria*: role of PknG. *BMC Microbiol.* 9:271. 10.1186/1471-2180-9-271 20030858 PMC2816201

[B9] Chen D. Ma S. He L. Yuan P. She Z. Lu Y. (2017). Sclerotiorin inhibits protein kinase G from *Mycobacterium tuberculosis* and impairs mycobacterial growth in macrophages. *Tuberculosis* 103 37–43. 10.1016/j.tube.2017.01.001 28237032

[B10] Chen J. W. Murphy T. L. Willingham M. C. Pastan I. August J. T. (1985). Identification of two lysosomal membrane glycoproteins. *J. Cell Biol.* 101 85–95. 10.1083/jcb.101.1.85 2409098 PMC2113627

[B11] Cooper J. A. (1987). Effects of cytochalasin and phalloidin on actin. *J. Cell Biol.* 105 1473–1478. 10.1083/jcb.105.4.1473 3312229 PMC2114638

[B12] Couldwell W. T. Hinton D. R. He S. Chen T. C. Sebat I. Weiss M. H. (1994). Protein kinase C inhibitors induce apoptosis in human malignant glioma cell lines. *FEBS Lett.* 345 43–46. 10.1016/0014-5793(94)00415-3 8194597

[B13] Cowley S. Ko M. Pick N. Chow R. Downing K. J. Gordhan B. G. (2004). The *Mycobacterium tuberculosis* protein serine/threonine kinase PknG is linked to cellular glutamate/glutamine levels and is important for growth in vivo. *Mol. Microbiol.* 52 1691–1702. 10.1111/j.1365-2958.2004.04085.x 15186418

[B14] Daigneault M. Preston J. A. Marriott H. M. Whyte M. K. Dockrell D. H. (2010). The identification of markers of macrophage differentiation in PMA-stimulated THP-1 cells and monocyte-derived macrophages. *PLoS One* 5:e8668. 10.1371/journal.pone.0008668 20084270 PMC2800192

[B15] Frieden T. R. Sterling T. R. Munsiff S. S. Watt C. J. Dye C. (2003). Tuberculosis. *Lancet* 362 887–899. 10.1016/S0140-6736(03)14333-4 13678977

[B16] Koul A. Choidas A. Tyagi A. K. Drlica K. Singh Y. Ullrich A. (2001). Serine/threonine protein kinase PknF and PknG of *Mycobacterium tuberculosis*: characterization and localization. *Microbiology* 147 2307–2314. 10.1099/00221287-147-8-2307 11496007

[B17] Matteelli A. Roggi A. Carvalho A. C. (2014). Extensively drug-resistant tuberculosis: epidemiology and management. *Clin. Epidemiol.* 6 111–118. 10.2147/CLEP.S35839 24729727 PMC3979688

[B18] Mosmann T. (1983). Rapid colorimetric assay for cellular growth and survival: application to proliferation and cytotoxicity assays. *J. Immunol. Methods* 65 55–63. 10.1016/0022-1759(83)90303-4 6606682

[B19] Scherr N. Honnappa S. Kunz G. Mueller P. Jayachandran R. Winkler F. (2007). Structural basis for the specific inhibition of protein kinase G, a virulence factor of *Mycobacterium tuberculosis*. *Proc. Natl. Acad. Sci. U.S.A.* 104 12151–12156. 10.1073/pnas.0702842104 17616581 PMC1924570

[B20] Singh N. Tiwari S. Srivastava K. K. Siddiqi M. I. (2015). Identification of novel inhibitors of *Mycobacterium tuberculosis* PknG using pharmacophore based virtual screening, docking, molecular dynamics simulation, and their biological evaluation. *J. Chem. Inf. Model.* 55 1120–1129. 10.1021/acs.jcim.5b00150 25965448

[B21] Sundaramurthy V. Korf H. Singla A. Scherr N. Nguyen L. Ferrari G. (2017). Survival of *Mycobacterium tuberculosis* and *Mycobacterium bovis* BCG in lysosomes in vivo. *Microbes Infect.* 19 515–526. 10.1016/j.micinf.2017.06.008 28689009

[B22] Sipos A. Pató J. Székely R. Hartkoorn R. C. Kékesi L. Õrfi L. (2015). Lead selection and characterization of antitubercular compounds using the Nested Chemical Library. *Tuberculosis* 95 (Suppl. 1), S200–S206. 10.1016/j.tube.2015.02.02 25801335

[B23] Smith D. B. Johnson K. S. (1988). Single-step purification of polypeptides expressed in *Escherichia coli* as fusions with glutathione S-transferase. *Gene* 67 31–40. 10.1016/0378-1119(88)90005-4 3047011

[B24] Székely R. Wáczek F. Szabadkai I. Németh G. Hegymegi-Barakonyi B. Eros D. (2008). A novel drug discovery concept for tuberculosis: inhibition of bacterial and host cell signalling. *Immunol. Lett.* 116 225–231. 10.1016/j.imlet.2007.12.005 18258308

[B25] Walburger A. Koul A. Ferrari G. Nguyen L. Prescianotto-Baschong C. Huygen K. (2004). Protein kinase G from pathogenic *Mycobacteria* promotes survival within macrophages. *Science* 304 1800–1804. 10.1126/science.1099384 15155913

[B26] Wang S. Midgley C. A. Scaërou F. Grabarek J. B. Griffiths G. Jackson W. (2010). Discovery of N-phenyl-4-(thiazol-5-yl) primidin-2-amine aurora kinase inhibitors. *J. Med. Chem.* 53 4367–4378. 10.1021/jm901913s 20462263

[B27] Weinblatt M. E. Kavanaugh A. Genovese M. C. Musser T. K. Grossbard E. B. Magilavy D. B. (2010). An oral spleen tyrosine kinase (Syk) inhibitor for rheumatoid arthritis. *N. Eng. J. Med.* 363 1303–1312. 10.1056/NEJMoa1000500 20879879

[B28] Zabludoff S. D. Deng C. Grondine M. R. Sheehy A. M. Ashwell S. Caleb B. L. (2008). AZD7762, a novel checkpoint kinase inhibitor, derives checkpoint abrogation and potentiates DNA-targeted therapies. *Mol. Cancer Ther.* 7 2955–2966. 10.1158/1535-7163.MCT-08-0492 18790776

[B29] Zumla A. George A. Sharma V. Herbert R. H. Baroness Masham of Ilton Oxley A. (2015). The WHO 2014 global tuberculosis report–further to go. *Lancet Glob. Health* 3 e10–e12. 10.1016/S2214-109X(14)70361-4 25539957

