# Supplementary figures and images for: Identification of Novel Mycobacterial Inhibitors Against Mycobacterial Protein Kinase G
Source: Front Microbiol. 2018 Jul 12;9:1517. doi: 10.3389/fmicb.2018.01517 (PMC6052090; doi:10.3389/fmicb.2018.01517)

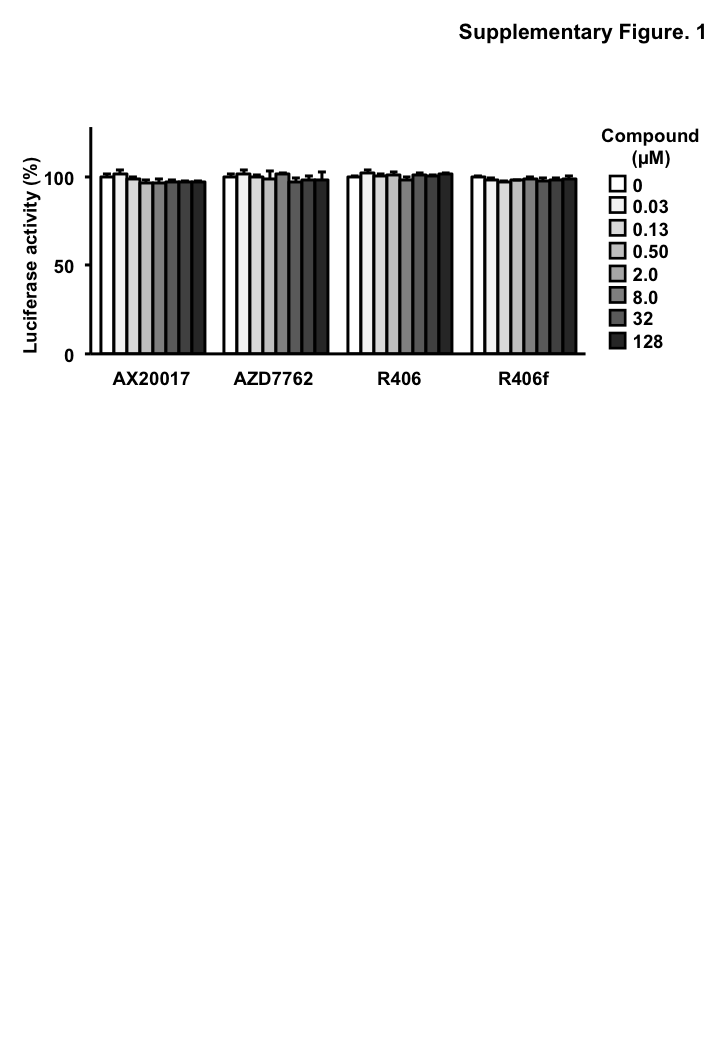

Supplement: FIGURE S1 — Effect of different compounds on luciferase activity. Luciferase activity was examined using 1 μM of ATP, fourfold serial dilutions of compounds in the range from 0.03 to 128 μM. R406f: R406-free base. [file Image_1.TIFF]
