# Supplementary material for: Identification of Novel Mycobacterial Inhibitors Against Mycobacterial Protein Kinase G
Source: Front Microbiol. 2018 Jul 12;9:1517. doi: 10.3389/fmicb.2018.01517 (PMC6052090; doi:10.3389/fmicb.2018.01517)
Supplement: TABLE S1 — Inhibitory activity of each PknG inhibitor by luciferase-based PknG kinase assay. [file Table_1.PDF]

Supplementary Table. 1

	Measurement 1	Measurement 2		Measurement 1	Measurement 2
ATP-/ PknG-	209	209	BS-181 hydrochloride	12,258	12,276
ATP+/ PknG-	94,439	97,225	Imatinib(STI571)	13,337	13,008
ATP+/ PknG+	15,538	14,596	Tandutinib (MLN518)	12,386	13,430
ATP+/ PknG+ (+AX20017)	59,213	57,250	SNS-314 Mesylate	13,992	14,967
Axitinib	12,811	10,491	PD318088	11,899	13,430
ZSTK474	13,992	10,882	Pazopanib Hydrochloride	12,649	13,600
XL147	13,896	10,876	GDG-0879	12,371	13,914
WZ4002	14,189	10,664	PLX-4720	17,135	18,789
TG100-115	19,680	14,147	PIK-75 Hydrochloride	19,273	19,584
HMN-214	14,895	10,320	VX-745	13,520	13,361
**AZD7762**	**45,018**	**49,923**	AT7519	13,609	13,295
PIK-293	14,530	15,164	AZD8330	13,878	13,397
Imatinib Mesylate	14,572	14,805	VX-702	12,993	12,386
BI 2536	13,780	15,547	ENMD-2076	18,056	17,963
LY2228820	11,821	11,531	BIRB 796	13,005	13,887
AZD6244 (Selumetinib)	12,093	10,395	PD0325901	13,068	13,869
SB 216763	14,124	11,247	LY294002	12,921	15,179
PD98059	12,913	10,386	Roscovitine(CYC202)	12,916	14,961
ON-01910	12,706	10,150	KU-0063794	13,968	14,566
PHA-793887	12,802	10,347	SP600125	20,407	22,129
**R406 free base**	**48,202**	**54,011**	Hesperadin	12,916	15,362
Indirubin	12,703	13,642	KW 2449	14,124	16,043
Motesanib Diphosphate	14,303	15,900	BIBF1120 (Vargatef)	12,518	14,130
XL880 (GSK1363089)	14,641	15,696	BMS-599626	13,445	13,149
XL765	11,080	10,168	Aurora A Inhibitor I	18,726	21,232
CI-1033 (Canertinib)	12,945	11,450	GSK461364	12,566	13,603
SB 202190	13,941	13,301	VX-680	13,433	14,201
SNS-032 (BMS-387032)	14,130	13,747	PHA-739358 (Danusertib)	16,435	18,146
BAY 73-4506 (Regorafenib)	14,255	13,786	TGX-221	14,207	14,112
AS-605240	14,052	12,222	WYE-354	14,784	15,427
PIK-93	13,182	13,502	AS703026	13,221	14,985
AZD8055	13,807	13,038	BIX 02188	12,919	14,470
Quercetin (Sophoretin)	14,106	13,863	Deforolimus (MK-8669)	13,639	13,098
Sunitinib Malate	17,458	16,151	SU11274 (PKI-SU11274)	14,581	14,614
Everolimus (RAD001)	13,256	11,696	AP24534	15,903	17,670
AC-220	12,464	10,664	**R406**	**48,896**	**53,469**
Erlotinib Hydrochloride	12,506	11,558	GDG-0941	17,171	18,071
ZM-447439	13,140	12,805	JNJ-38877605	13,899	15,254
AZD1152-HQPA (Barasertib)	14,746	15,086	**CYC116**	**44,578**	**46,966**
PIK-90	12,521	12,802	IC-87114 (PIK-293)	14,626	15,254
PHA-680632	14,067	13,188	SB 525334	13,421	15,385
CCT129202	13,098	13,080	BIX 02189	12,901	14,476
